# 15-PGDH inhibition activates the splenic niche to promote hematopoietic regeneration

**DOI:** 10.1172/jci.insight.143658

**Published:** 2021-03-22

**Authors:** Julianne N.P. Smith, Dawn M. Dawson, Kelsey F. Christo, Alvin P. Jogasuria, Mark J. Cameron, Monika I. Antczak, Joseph M. Ready, Stanton L. Gerson, Sanford D. Markowitz, Amar B. Desai

**Affiliations:** 1 Department of Medicine and Case Comprehensive Cancer Center Case Western Reserve University, Cleveland, Ohio, USA.; 2 Department of Biochemistry, University of Texas Southwestern Medical Center, Dallas, Texas, USA.; 3 Simmons Cancer Center, University of Texas Southwestern Medical Center, Dallas, Texas, USA.; 4 University Hospitals Seidman Cancer Center, Cleveland, Ohio, USA.

**Keywords:** Hematology, Eicosanoids, Hematopoietic stem cells

## Abstract

The splenic microenvironment regulates hematopoietic stem and progenitor cell (HSPC) function, particularly during demand-adapted hematopoiesis; however, practical strategies to enhance splenic support of transplanted HSPCs have proved elusive. We have previously demonstrated that inhibiting 15-hydroxyprostaglandin dehydrogenase (15-PGDH), using the small molecule (+)SW033291 (PGDHi), increases BM prostaglandin E_2_ (PGE_2_) levels, expands HSPC numbers, and accelerates hematologic reconstitution after BM transplantation (BMT) in mice. Here we demonstrate that the splenic microenvironment, specifically 15-PGDH high-expressing macrophages, megakaryocytes (MKs), and mast cells (MCs), regulates steady-state hematopoiesis and potentiates recovery after BMT. Notably, PGDHi-induced neutrophil, platelet, and HSPC recovery were highly attenuated in splenectomized mice. PGDHi induced nonpathologic splenic extramedullary hematopoiesis at steady state, and pretransplant PGDHi enhanced the homing of transplanted cells to the spleen. 15-PGDH enzymatic activity localized specifically to macrophages, MK lineage cells, and MCs, identifying these cell types as likely coordinating the impact of PGDHi on splenic HSPCs. These findings suggest that 15-PGDH expression marks HSC niche cell types that regulate hematopoietic regeneration. Therefore, PGDHi provides a well-tolerated strategy to therapeutically target multiple HSC niches, promote hematopoietic regeneration, and improve clinical outcomes of BMT.

## Introduction

The spleen influences hematopoietic stem cell transplantation outcomes, yet the mechanisms regulating splenic hematopoiesis posttransplantation are not well understood. In mice, transplanted hematopoietic stem and progenitor cells (HSPCs) home to the spleen prior to the BM (1), and spleen-homed HSPCs demonstrate superior function relative to BM-homed HSPCs several hours after transplant (2). In the days to weeks after transplantation, hematopoietic foci form in the spleen (3, 4), corresponding to sites of HSPC proliferation and maturation. In humans, splenomegaly portends delayed neutrophil and platelet engraftment (5); however, splenectomy does not improve survival and has been linked to graft versus host disease and lymphoproliferative disease (6, 7). Thus, the spleen is capable of both positively and negatively regulating hematopoietic reconstitution, and further investigation into the interactions between the splenic microenvironment and transplanted HSPCs is necessary.

HSPCs lodge in the spleen via CXCL12 expressed by perisinusoidal cells in the red pulp (8). In the setting of splenic extramedullary hematopoiesis (EMH), CXCL12^+^ and stem cell factor–positive (SCF^+^) stromal cell populations also promote HSPC activation and myeloerythroid progenitor cell expansion (9), whereas VCAM1^+^ macrophages retain HSPCs in the spleen (10). These findings highlight the potential therapeutic utility of strategies to promote or limit EMH, to improve hematopoietic regeneration or limit inflammation mediated by spleen-derived myeloid cells, as occurs in cardiovascular and neurovascular disease (4, 11, 12).

We have previously shown that inhibition of 15-hydroxyprostaglandin dehydrogenase (15-PGDH) expands HSPCs at steady state and enhances hematopoietic regeneration after transplantation and during BM failure (13–15). 15-PGDH inhibition (PGDHi) increases PGE_2_ and induces *Cxcl12* and *Scf* expression by BM stromal cells; however, the impact of PGDHi on the spleen, particularly after transplant, is not well understood. Here, we identify splenic 15-PGDH and, specifically, 15-PGDH–expressing macrophages, megakaryocytes (MKs), and mast cells (MCs) as regulators of EMH and propose that targeting splenic 15-PGDH prior to transplant will enhance homing and regeneration, resulting in improved clinical outcomes.

## Results

### The spleen was critical for PGDHi-mediated hematopoietic regeneration.

**Figure 1:**
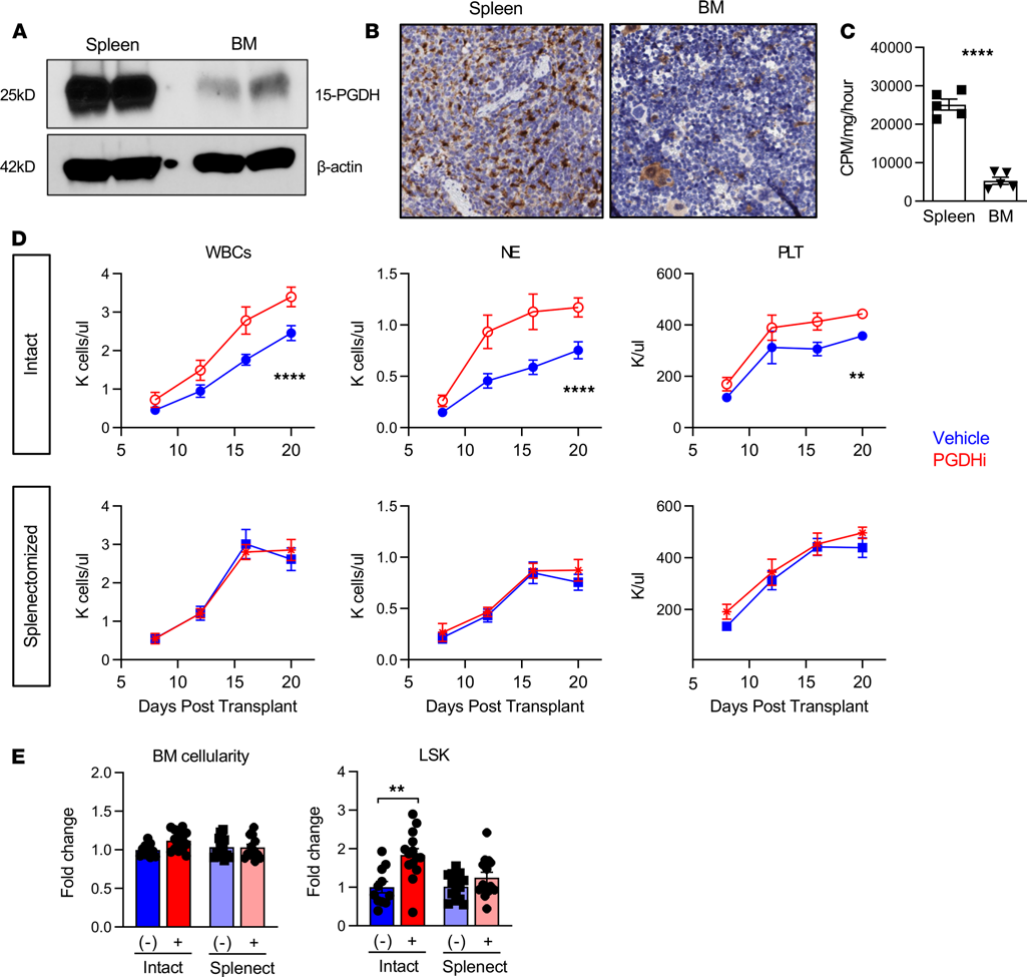
The spleen is critical for PGDHi-mediated hematopoietic regeneration. (**A**) Representative detection of 15-hydroxyprostaglandin dehydrogenase (15-PGDH) at 25 kDa and β-actin at 42 kDa in splenocyte and BM cell lysates. Two independent experiments of *n* = 2 mice per experiment. (**B**) Representative images of 15-PGDH staining (brown) in splenic red pulp (left) and tibial BM core (right). (Original magnification, ×20.) Three independent experiments of *n* = 2 mice per experiment. (**C**) Quantification of 15-PGDH enzymatic activity in spleen and BM, expressed as cpm per mg total protein, per hour. *n* = 5 mice. Error bars represent SEM. (**D**) Peripheral WBC, neutrophil (NE), and platelet (PLT) recovery in intact (top) and splenectomized (bottom) transplant recipients treated with either vehicle (Veh; blue) or 15-PGDH inhibitor (PGDHi; red). *n* = 12–15 mice/group. Data represent mean ± SEM. (**E**) BM cellularity and quantification of lineage^–^c-Kit^+^Sca-1^+^ (LSK) cells per hind limb of control and splenectomized recipients 20 days after transplant, treated with Veh (-) or PGDHi (+), expressed as fold change. *n* = 11–14 mice per group. Error bars represent SEM. **P < 0.01, ****P < 0.0001. Student’s *t* test used for all except peripheral blood recovery, where 2-way ANOVA was used and asterisks denote Veh vs. PGDHi over days 7 through 20.

To determine if the spleen responds to 15-PGDH inhibition, we examined 15-PGDH expression levels in splenic tissue. Splenic 15-PGDH expression was substantially elevated versus that of BM (Figure 1A). IHC staining for 15-PGDH also revealed a striking difference in the abundance of 15-PGDH^+^ cells (Figure 1B). Whereas the BM displayed relatively rare 15-PGDH^+^ cells, which comprised smaller hematopoietic cells and MKs, splenic 15-PGDH^+^ cells were highly numerous, particularly within the red pulp (Supplemental Figure 1; supplemental material available online with this article; https://doi.org/10.1172/jci.insight.143658DS1). Consistent with these findings, 15-PGDH enzymatic activity was significantly higher in splenic compared with BM cell lysates (Figure 1C), demonstrating that the abundant 15-PGDH is enzymatically active. Together these results suggested that the spleen may be more sensitive than the marrow to pharmacologic 15-PGDH targeting.

Having established that 15-PGDH is expressed much more highly in the spleen than the marrow, we next sought to determine whether the spleen is required for the hematopoietic protective effects of 15-PGDH inhibition (PGDHi; ref. 14). To test this, we compared short-term hematologic recovery from transplant in splenectomized versus intact mice. Although vehicle-treated splenectomized mice recovered blood counts slightly faster than intact controls, as has been reported (16, 17), splenectomy markedly attenuated the impact of PGDHi on neutrophil recovery and abrogated the impact of PGDHi on platelet recovery (Figure 1D and Supplemental Figure 2). Importantly, PGDHi-treated mice with spleens reached absolute neutrophil counts of 935 by day 12, compared with 456 in splenectomized counterparts. Splenectomized mice also failed to show a PGDHi-dependent acceleration of total WBC recovery, suggesting that myeloid to lymphoid lineage skewing was not occurring. In addition, PGDHi did not enhance donor-derived HSPC numbers in the BM of splenectomized mice on day 20 (Figure 1E and Supplemental Figure 3). These data therefore establish that the spleen was required for PGDHi-mediated hematologic recovery.

### PGDHi induced splenic EMH.

**Figure 2:**
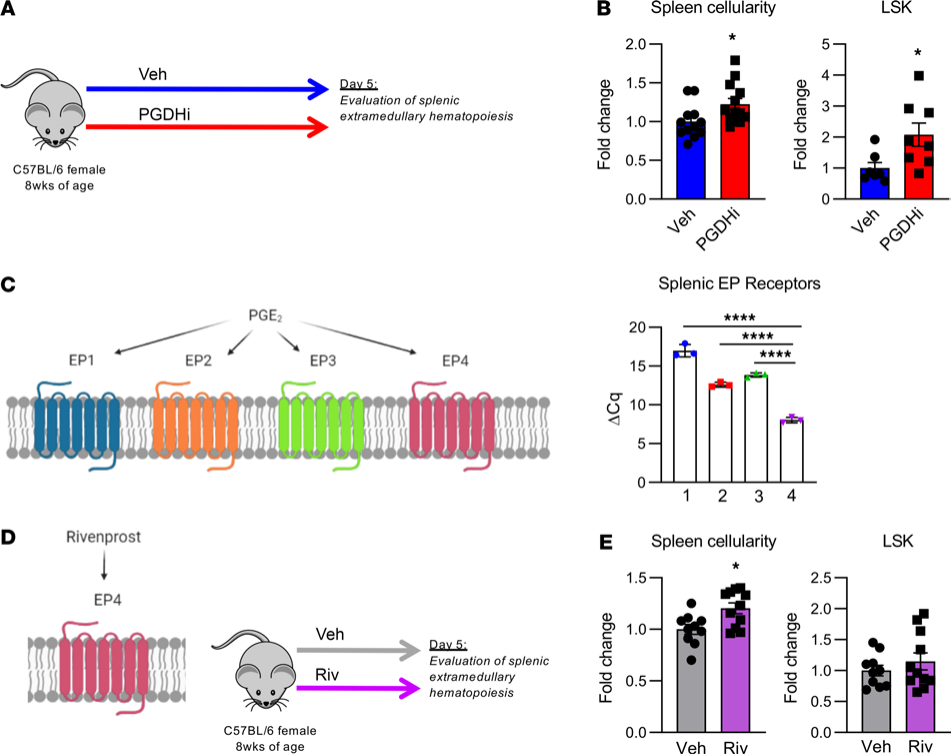
PGDHi induces splenic extramedullary hematopoiesis. (**A**) Schematic depicting 15-hydroxyprostaglandin dehydrogenase (15-PGDH) inhibition (PGDHi) in steady-state mice over the course of 5 days (9 injections). (**B**) Quantification of splenic cellularity and lineage^–^c-Kit^+^Sca-1^+^ (LSK) cells per spleen after 5 days vehicle (Veh) and PGDHi treatment, expressed as fold change. *n* = 12–13 mice/group for splenic cellularity and *n* = 7–8 mice per group for splenic LSK number. Error bars represent SEM. (**C**) EP1–4 (*Ptger1*, *2*, *3*, and *4*) expressed as ΔCq relative to *B2m* control gene expression levels in CD45^+^ splenocytes. *n* = 3 mice. Error bars represent SEM. (**D**) Schematic depicting Rivenprost (Riv) administration in mice over the course of 5 days (9 doses). (**E**) Quantification of splenic cellularity and LSK numbers after 5 days’ Veh and Riv treatment, expressed as fold change. *n* = 10–11 mice/group. Error bars represent SEM. *P < 0.05, ****P < 0.0001. Student’s *t* test used for all except for splenic EP receptor expression, where 1-way ANOVA with Tukey’s multiple comparisons test was used. PGE_2_ signaling diagrams created with BioRender.com.

To determine if an increase in splenic EMH may underlie PGDHi-mediated hematopoietic protection after transplant, and thus explain why splenectomized mice do not respond to PGDHi, we characterized the spleens of healthy mice treated for 5 days with PGDHi (Figure 2A). PGDHi-treated mice showed significant increases in total splenic cellularity and in splenic HSPCs (Figure 2B), suggesting that at homeostasis, 15-PGDH negatively regulated hematopoiesis in the spleen. PGDHi also increases BM HSPCs at homeostasis (14); however, PGDHi did not significantly increase circulating HSPCs (Supplemental Figure 4), and therefore it is unlikely that HSPC mobilization from the BM to the spleen accounted for this effect.

PGE2 signals via prostaglandin receptors EP1–4 (18). Analysis of EP1–4 expression in splenic CD45^+^ cells revealed a significant predominance in the expression of the gene encoding EP4 relative to EP1, 2, and 3 (Figure 2C). To determine if PGE2 signaling via EP4 may underlie the PGDHi-induced EMH, we treated mice with the EP4-specific agonist, Rivenprost (19) (Figure 2D). Although EP4 agonism was sufficient to increase splenic cellularity, it failed to significantly expand splenic HSPCs (Figure 2E). These data therefore indicate that PGDHi mediated splenic EMH in part via the actions of PGE2-EP4 signaling but do not rule out involvement of EP1–3 and may indicate that additional pathways, outside of EP signaling, may be involved in PGDHi-mediated regeneration.

### PGDHi expanded functional HSPCs in the spleen.

**Figure 3:**
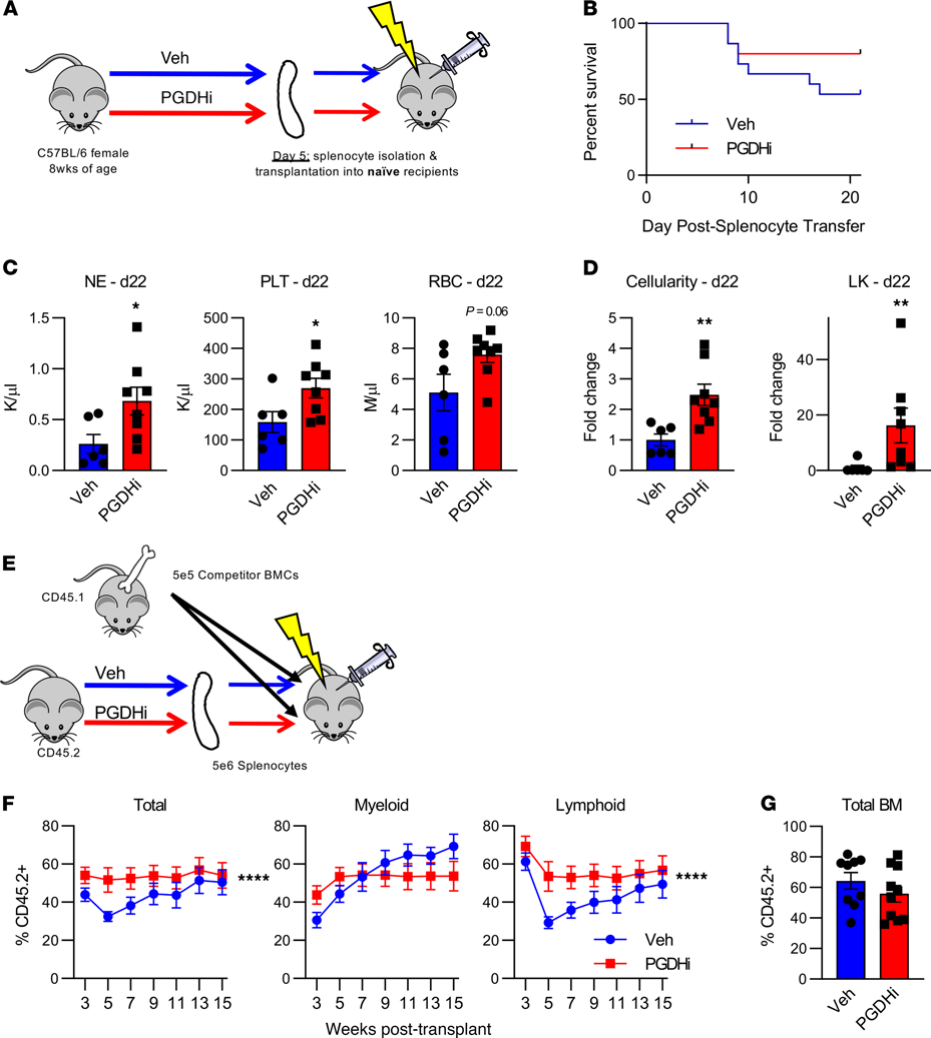
PGDHi expands functional hematopoietic stem and progenitor cells in the spleen. (**A**) Schematic depicting the transplantation of splenocytes from PGDHi-treated or vehicle-treated (Veh-treated) donors into irradiated, untreated recipients. (**B**) Overall survival time of mice that received splenocytes from Veh-treated or PGDHi-treated donors. *n* = 16 mice/group. Statistical testing by log rank (Mantel-Cox) test. (**C**) Quantification of peripheral blood neutrophils (NE), platelets (PLT), and RBCs in mice that received splenocytes from either Veh-treated or PGDHi-treated donors, 22 days after transplant. *n* = 6–8 mice/group. Error bars represent SEM. (**D**) Quantification of BM cellularity and lineage^–^c-Kit^+^ (LK) BM cells in recipient mice, 22 days after transplant, expressed as fold change. *n* = 6–8 mice/group. Error bars represent SEM. (**E**) Schematic depicting the competitive transplantation of splenocytes from PGDHi-treated or Veh-treated donors together with BM from untreated CD45.1^+^ donors into irradiated, untreated recipients. (**F**) PB chimerism at indicated time points after transplant, as measured by the percent CD45.2^+^ cells in total PB, CD11b^+^ Myeloid PB, and B220^+^/CD3ε^+^ lymphoid PB. *n* = 9–11 recipients/group. Data represent mean ± SEM. (**G**) Total BM cell chimerism at 15 weeks after transplant, as measured by the percentage of CD45.2^+^ cells. *n* = 9–11 recipients/group. Error bars represent SEM. *P < 0.05, **P < 0.01. Statistical testing of (**C** and **D**) was done by Student’s *t* test, except in the case of LK cell fold change, where a Mann-Whitney *U* test was performed. Statistical testing of (**F**) was done by Student’s *t* test of the area under the curve of Veh vs. PGDHi curves.

To test whether splenic EMH corresponded to an increase in functional HSPCs in the spleen of PGDHi-treated mice, we transplanted splenocytes from PGDHi-treated donors into lethally irradiated recipients (Figure 3A). A limiting cell dose of 2 × 10^6^ splenocytes was chosen to assess both survival and hematologic recovery. Of the mice that received control splenocytes, 47% succumbed to hematopoietic failure (Figure 3B), evidenced by pallor, hypothermia, and lethargy (not shown). In contrast, splenocytes derived from PGDHi-treated donors conferred 80% survival. To assess hematologic recovery, surviving mice were sacrificed 22 days after transplant. Recipients of splenocytes from PGDHi donors showed marked increases in peripheral blood neutrophils, platelets, and a trend toward increased RBCs (Figure 3C). Although the BM remained hypocellular, PGDHi donor splenocytes were associated with significantly increased engraftment of the BM in total and the lineage^–^c-Kit^+^ immature compartment specifically (Figure 3D). To evaluate the impact of PGDHi on the long-term BM engraftment capacity of splenic HSPCs, recipient mice were also analyzed 70 days after transplant. At this time point, mice were no longer pancytopenic, although they did exhibit persistent thrombocytopenia, which was less severe in recipients of PGDHi donor cells (Supplemental Figure 5A). Moreover, mice that received splenocytes from PGDHi-treated donors maintained an increase in BM cellularity, and further displayed a significant increase in the LSK CD48^–^ CD150^–^ marked short-term HSC population but did not exhibit differences in phenotypic long-term numbers (Supplemental Figure 5B). Together these data demonstrate that PGDHi enhanced the hematopoietic capacity of the spleen to increase cellular proliferation and expanded a population of functionally active spleen-resident HSPCs.

Having established that PGDHi elicited splenic EMH, we next questioned whether drug treatment acted specifically on splenic HSPCs with short-term or lineage-biased repopulation activity. To address this, we competitively transplanted splenocytes from PGDHi-treated and vehicle-treated donors with competitor whole BM cells and quantified peripheral blood chimerism 3–15 weeks after transplant (Figure 3E). PGDHi conferred a repopulation advantage to transplanted splenocytes over the course of the transplant (Figure 3F). Specifically, splenocytes from PGDHi-treated donors gave rise to increased myeloid lineage cells 3 weeks after transplant and subsequently increased lymphoid cells 5–9 weeks after transplant, while demonstrating no change in week 15 BM chimerism (Figure 3G), thus demonstrating that PGDHi-induced splenic EMH occurred via actions on HSPCs with multilineage repopulating activity.

### Recipient PGDHi preconditioning enhanced homing to the BM and splenic niches.

**Figure 4:**
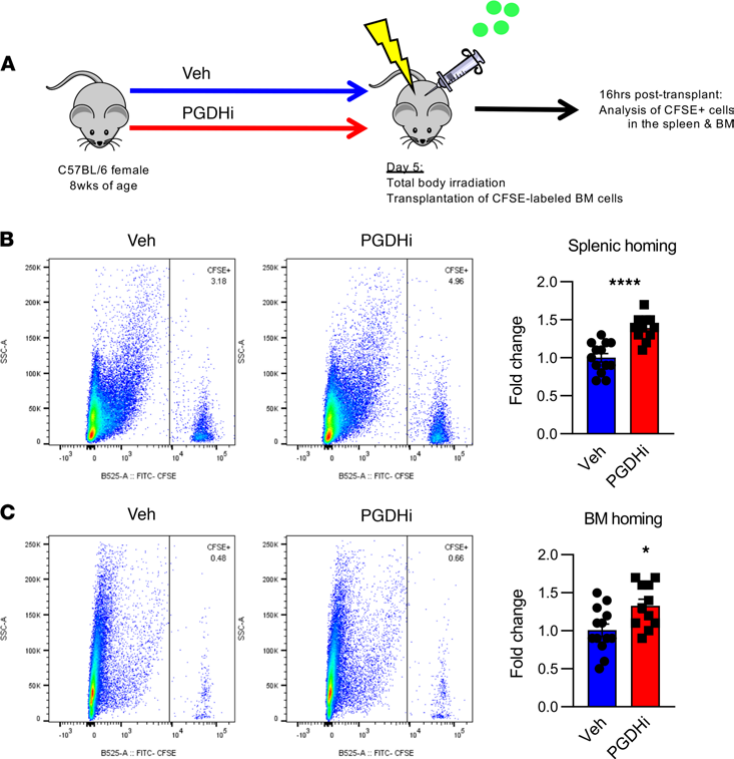
Recipient PGDHi preconditioning enhances homing to the BM and splenic niches. (**A**) Schematic depicting the analysis of homing into PGDHi-pretreated recipients. (**B**) Representative flow cytometry plots depicting the detection of CFSE^+^ cells among total splenocytes isolated 16 hours after transplantation of pretreated mice. Graph represents fold change in the frequency of CFSE^+^ splenocytes. *n* = 11–13 mice/group. Error bars represent SEM. (**C**) Representative flow cytometry plots depicting the detection of CFSE^+^ cells among total BM cells isolated 16 hours after transplant in vehicle-pretreated and PGDHi-pretreated mice. Graph represents fold change in the frequency of CFSE^+^ BM cells. *n* = 11–13 mice/group. Error bars represent SEM. *P < 0.05, ****P < 0.0001. Statistical testing by Student’s *t* test.

Much like the BM, splenic hematopoiesis is regulated through the local tissue microenvironment (9). Because PGDHi elicited splenic EMH in healthy mice, we next sought to test the therapeutic relevance of these findings and determine if pretransplant PGDHi would increase splenic homing (Figure 4A). Recipient mice treated with PGDHi prior to transplant demonstrated a 1.5-fold increase in the frequency of donor cells present in the spleen 16 hours after transplant (Figure 4B). Pretransplant PGDHi also increased the homing of transplanted cells to the BM (Figure 4C), suggesting that 15-PGDH inhibition enhanced the capacity of both the splenic and the BM microenvironment to recruit and support engrafting cells.

### PGDHi elicited a prohematopoietic gene expression signature in the spleen.

**Figure 5:**
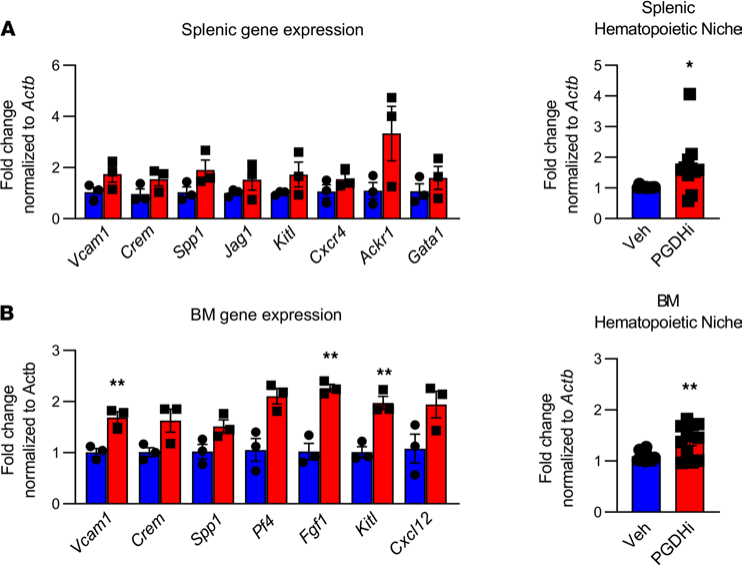
PGDHi elicits a prohematopoietic gene expression signature in the spleen and BM. (**A**) Relative expression of indicated genes in lymphoid-depleted splenocytes from vehicle-treated (blue) and PGDHi-treated (red) mice, normalized to *Actb* (left). Fold change in the expression of all hematopoietic niche-related genes, listed at right. *n* = 3 mice/group. Error bars represent SEM. (**B**) Relative expression of indicated genes in lymphoid-depleted BM cells from vehicle-treated (blue) and PGDHi-treated (red) mice, normalized to *Actb* (left). Fold change in the expression of all hematopoietic niche-related genes, listed in **A** (right). *n* = 3 mice/group. Error bars represent SEM. *P = 0.02, **P < 0.005. Statistical testing by Student’s *t* test.

We next sought to identify whether these PGDHi-mediated effects were associated with a prohematopoietic gene expression signature in the spleen and BM. Because 15-PGDH expression localized to the splenic red pulp, we analyzed the expression of a number of hematopoietic niche-related genes (20–22) (Table 1) in lymphoid-depleted BM and splenic cells after 5 days of vehicle or PGDHi treatment. Consistent with our findings that PGDHi induced splenic EMH and promoted homing to the splenic and BM niches, we found that a number of factors were modestly induced including the niche retentive factors *Spp1* and *Vcam1* (10, 23) and the quiescence-promoting factor *Kitl* (24) (Figure 5, A and B). PGDHi elicited moderate induction of the atypical chemokine receptor 1 (*Ackr1*) gene, which has been implicated in maintaining hematopoietic quiescence via the macrophage niche (25), specifically in the spleen (Supplemental Figure 6). The sum of the individual gene expression changes across the panel of hematopoietic niche–associated factors revealed a marked increase in both organs, however, suggesting that PGDHi induced a pro-niche response that facilitated post–hematopoietic stem cell transplant engraftment.

### 15-PGDH was highly enriched in splenic and BM MCs, MKs, and macrophages.

**Figure 6:**
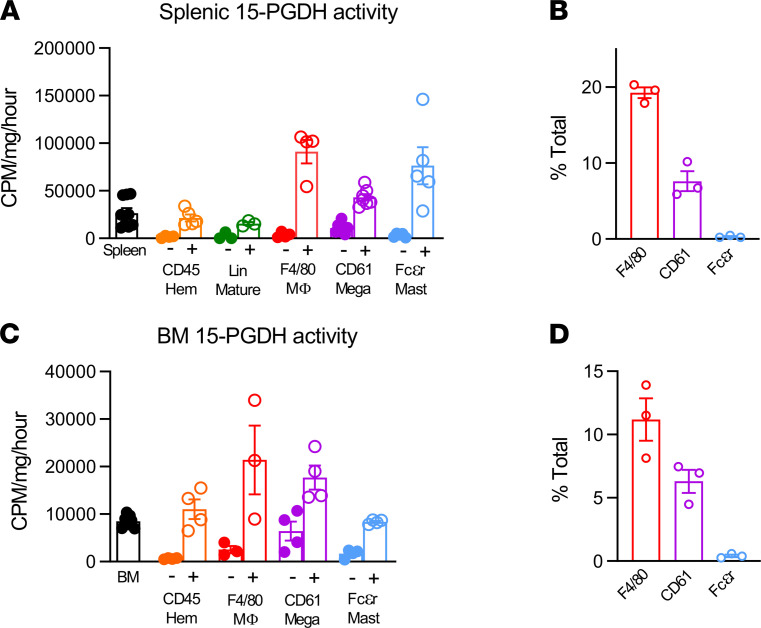
15-PGDH activity is highly enriched in splenic and marrow mast cells, megakaryocytes, and macrophages. (**A**) Quantification of 15-hydroxyprostaglandin dehydrogenase (15-PGDH) enzymatic activity in total unfractionated spleen and in splenic CD45, hematopoietic lineage (Lin), F4/80, CD61, and Fcεr1a negative and positive fractions, respectively, expressed as cpm per mg total protein, per hour. Filled symbols indicate each marker’s negative population. *n* = 3–7 mice/cell population. Error bars represent SEM. (**B**) Quantification of the frequency of F4/80^+^, CD61^+^, and Fcεr1a^+^ cells in the murine spleen. *n* = 3 mice. Error bars represent SEM. (**C**) Quantification of 15-PGDH enzymatic activity in total unfractionated BM and in BM CD45, F4/80, CD61, and Fcεr1a negative and positive fractions, respectively, expressed as cpm/mg total protein/hr. Filled symbols indicate each marker’s negative population. *n* = 3–7 mice/cell population. Error bars represent SEM. (**D**) Quantification of the frequency of F4/80^+^, CD61^+^, and Fcεr1a^+^ cells in the murine BM. *n* = 3 mice. Error bars represent SEM.

Given the functional significance of splenic 15-PGDH, we sought to identify the cellular sources of 15-PGDH activity in murine spleen. IHC 15-PGDH staining principally identified hematopoietic cell types, including MKs, whereas splenic stroma revealed very low 15-PGDH activity (data not shown). Isolation of bulk CD45^+^ hematopoietic cells showed no enrichment of 15-PGDH enzymatic activity per milligram protein compared with total unfractionated splenocytes (Figure 6A). To determine whether immature hematopoietic cells are 15-PGDH^+^, and thus direct targets of PGDHi, we compared hematopoietic lineage-negative cells with lineage-positive cells. 15-PGDH activity per milligram protein was very low in the immature cell fraction compared with the mature cell fraction, suggesting that 15-PGDH localized to a subset of mature cells. Analysis of myeloid cells by CD11b fractionation demonstrated relative enrichment (Supplemental Figure 7), and thus we reasoned that the major cellular sources of 15-PGDH included a myeloid cell type. Because prostanoid signaling is known to regulate macrophages, MKs, and MCs, we measured activity specifically within these fractions. F4/80^+^ macrophages accounted for the highest level of enzyme activity per milligram protein, but substantial enrichment was also measured in CD61^+^ MKs and FcεR1a^+^ MCs. Among these cell types, F4/80^+^ cells were the most numerous in the spleen, accounting for 19.3% of nucleated splenocytes (Figure 6B). In contrast, CD61^+^ and FcεR1a^+^ cells represented 7.6% and 0.3% of nucleated splenocytes, respectively. As a reference, CD3^+^ cells represented 28.2% of cells analyzed (data not shown). Notably, 15-PGDH activity also localized to CD61^+^, FcεR1a^+^, and F4/80^+^ cells in the BM (Figure 6C), though these levels were much lower than those of the corresponding splenic populations. Among 15-PGDH^+^ cell types, F4/80^+^ cells were also the most numerous in the BM (Figure 6D). These data therefore implicate splenic macrophages as the predominant cellular targets of PGDHi treatment in hematopoietic tissue.

### 15-PGDH localization and enzymatic activity was conserved in human BM.

**Figure 7:**
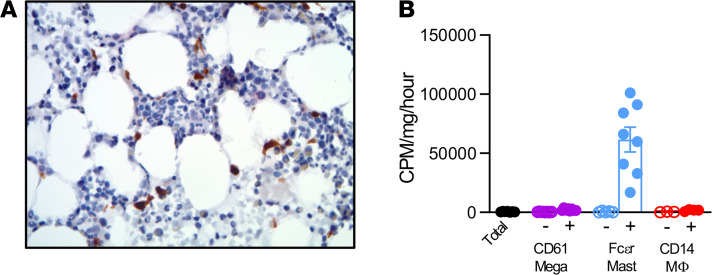
15-PGDH localization and enzymatic activity is conserved in human BM. (**A**) Representative image of 15-hydroxyprostaglandin dehydrogenase (15-PGDH) staining (brown) in a human BM biopsy. (Original magnification, ×40.) Six independent experiments of *n* = 1 marrow donor per experiment. (**B**) Quantification of 15-PGDH enzymatic activity in total (unfractionated) human BM compared with CD61, Fcεr1a, and CD14 negative and positive fractions, expressed as cpm/mg total protein/hr. Open symbols indicate each marker’s negative population. *n* = 3–5 donors. Error bars represent SEM.

To determine if 15-PGDH expression patterns are conserved between murine and human hematopoietic tissue, we evaluated healthy human biopsies and aspirates. 15-PGDH^+^ marrow cells were readily detectable in all human biopsies examined (Figure 7A). Positive cells varied in size and morphology and included pyramidal, elongated, and round cells. To identify the cellular localization of 15-PGDH activity, we separated cells from human BM aspirates on the basis of surface marker expression. Relative to the activity of total BM, FcεR1a^+^ MCs demonstrated 115-fold higher levels of specific 15-PGDH activity (Figure 7B). CD14^+^ macrophages and CD61^+^ MK lineage cells also demonstrated enzyme activity enrichment, though to lesser degrees than FcεR1a^+^ cells. These results establish that FcεR1a^+^ MCs, CD61^+^ MKs, and CD14^+^ macrophages were robust sources of 15-PGDH activity in human marrow and thus may comprise a therapeutically targetable human HSC niche.

## Discussion

Previously, we established that 15-PGDH regulates hematopoietic, colonic epithelial, and hepatic tissue regeneration (13–15). Pharmacologic 15-PGDH inhibition or loss of *Hpgd* expression (that encodes 15-PGDH) elevated BM prostaglandin E_2_, D_2_, and F_2_a levels; increased peripheral neutrophils; and expanded the BM HSPC compartment. PGDHi also enhanced the progenitor activity, homing, and reconstituting ability of murine BM, human BM, and umbilical cord blood. PGDHi induced the expression of niche factors in the BM. However, because splenic colony forming units and splenic HSPCs were also increased, we hypothesized that splenic 15-PGDH also negatively regulates hematopoietic regeneration. Here, we demonstrate robust 15-PGDH expression in splenic red pulp, which localized to macrophages, MKs, and MCs. Our observation that the spleen was required for PGDHi responses after transplant advances current understanding of splenic EMH and identifies potential therapeutic targets within the splenic HSPC niche.

PGE_2_ is an arachidonic acid derivative capable of increasing HSPC numbers in vivo and in vitro (26–28). Although PGE_2_ can be produced by a multitude of cell types, osteoblasts, endothelial cells, and monocyte/macrophage lineage cells have been most extensively characterized in the HSPC microenvironment (reviewed in ref. 29). HSPCs and nonhematopoietic microenvironmental cell types express PGE_2_ receptors (30), and agonism of EP2 and EP4 receptors on HSPCs activates Wnt signaling and increases the expression of antiapoptotic and proproliferative gene programs (31). PGE_2_ stimulation also increases HSPC CXCR4 expression (32), thus enhancing homing capacity. Recently, a role for macrophage-derived PGE_2_ in facilitating erythropoietin-induced erythropoiesis was reported by Chen et al. (33). Clinical trials have evaluated the ex vivo stimulation of human cord blood with the long-acting PGE_2_ analog dimethyl-PGE_2_ (dmPGE_2_) as a strategy to enhance engraftment (34). Ex vivo stimulation avoids the potential for off-target dmPGE_2_-induced toxicity; however, our data, together with the radioprotective and erythropoiesis-promoting effects of PGE_2_ (26, 27, 33), demonstrate the potential for additional benefit from strategies to elevate tissue PGE_2_ levels in transplant recipients. Future studies to evaluate dmPGE_2_ ex vivo graft stimulation combined with pretransplant PGDHi recipient conditioning are therefore warranted.

In the adult, hematopoiesis takes place primarily in the BM, where HSPCs are regulated by perivascular stromal cells, endothelial cells, macrophages, and MKs (35–39). The red pulp of the spleen serves as an alternative HSPC microenvironment when the BM is dysfunctional (reviewed in ref. 40), however, and provides myelopoiesis and erythropoiesis in response to infection (41), inflammation (42), and physical and psychological stress (43, 44). Rare HSPCs are found in murine spleen under homeostatic conditions (9), however, and recent reports demonstrate human splenic EMH in the absence of disease (45). Here we establish that PGDHi induced nonpathologic splenic EMH. Splenic endothelial and Tcf21^+^ stromal cells were recently shown to regulate EMH and particularly myeloerythroid lineage differentiation (9). Our studies do not directly address the impact of PGDHi on spleen stroma, but 15-PGDH activity was very low in splenic CD45^–^ cells, indicating that stromal cells were not likely direct PGDHi targets.

PGDHi likely increased splenic cellularity via PGE2 stimulation of EP4 receptor, as EP4-specific agonism recapitulates this phenotype. EP4 agonism is not sufficient to expand the pool of splenic HSPCs, however, suggesting activation of additional EP receptors was required for the induction of splenic EMH by PGDHi. Moreover, PGDHi potentiated splenic homing of transplanted cells. As the spleen is associated with delayed engraftment in some transplant patients (5), our data suggest that PGDHi may provide an alternative to splenectomy or splenic irradiation. Whether PGDHi improves hematopoietic function in other pathophysiologic states that involve splenic EMH, such as infection or blood loss, is an intriguing question.

BM MKs enforce HSC quiescence via CXCL4 and TGF-β but take on an FGF1-dependent HSC-activating role upon hematologic stress (38, 39). Similarly, macrophages maintain quiescence and niche retention at steady state in part through activities of *Ackr1* (25) and VCAM1 (ref. 10 and reviewed in ref. 46), but exacerbate inflammation and regulate HSPC differentiation in pathologic conditions (47). Our finding that splenic MKs and macrophages expressed high levels of enzymatically active 15-PGDH suggests that these cell types participated in the PGDHi-dependent regulation of splenic EMH. PGE_2_ limits inflammation in some contexts (48), and irradiation potentiates the inflammatory state of macrophages (49), and thus it is possible that PGDHi attenuates macrophage activation to preserve splenic niche function. Additionally, PGE2 inhibits TGF-β signaling (50); therefore, PGDHi treatment may modulate the role of splenic MKs from promoting HSC quiescence to activation. Our work also implicates MCs as components of the splenic EMH microenvironment. MCs are rich in histamine-containing and leukotriene-containing granules, however, and thus are poised to rapidly regulate the local tissue microenvironment. Moreover, leukotriene B_4_ has been hypothesized to promote HSPC differentiation at the expense of self-renewal (29), and PGE_2_ suppresses MC degranulation in anaphylaxis (51). Increased splenic myelopoiesis and thrombopoiesis have also been observed in MC-deficient mice (52). Future studies to evaluate the impact of PGDHi specifically on splenic MCs, MKs, and macrophages are warranted.

In conclusion, 15-PGDH was highly expressed and enzymatically active in the murine spleen. Pretransplant PGDHi induced a pro-niche gene signature in the splenic and BM microenvironments and induced splenic EMH, which translates to an increase in the homing of transplanted cells to the spleen. We find that the spleen was required for PGDHi-mediated leukocyte and platelet reconstitution and BM HSPC engraftment. This likely owes to a network of 15-PGDH^+^ macrophages, MKs, and rare MCs in the spleen. Therefore, our work identifies a pharmacologic strategy and the corresponding cellular targets that regulate extramedullary hematopoiesis. Small molecule 15-PGDH inhibition represents a therapeutic strategy to utilize the splenic microenvironment after transplant and likely in other disease states where rapid hematopoietic regeneration is needed.

## Methods

### Reagents.

15-PGDH inhibitors (+)SW033291 and (+)SW209415 were previously described (13, 14) and provided in-house. (+)SW033291 was prepared in a vehicle of 10% ethanol, 5% Cremophor EL, 85% dextrose-5 water, at 125 μg/200 μL for use at 5 mg/kg for a 25 g mouse, and administered i.p., twice per day, 6–8 hours apart. (+)SW209415 was prepared as previously described (13) and administered i.p. at 2.5 mg/kg, twice per day. Rivenprost (Cayman Chemical) was prepared in a vehicle of PBS for use at 30 μg/kg and administered i.p., twice per day, 6–8 hours apart. CFSE Cell Trace was purchased from Invitrogen, Thermo Fisher Scientific.

### Animals.

Steady-state and transplantation analyses were performed on 8-week-old female C57BL/6J mice obtained from The Jackson Laboratories. B6.SJL-*Ptprc^a^ Pepc^b^*/BoyJ and splenectomized C57BL/6 mice were obtained from The Jackson Laboratories. All animals were observed daily for signs of illness. Mice were housed in standard microisolator cages and maintained on a defined, irradiated diet and autoclaved water.

### Western blotting.

Cells were lysed using RIPA lysis buffer containing protease inhibitors. Lysates were centrifuged 9391*g* for 10 minutes at 4°C. Protein concentrations were determined by BCA assay. Proteins were separated using 4%–12% SDS-PAGE gels, then transferred to PVDF membranes, and probed with antibodies recognizing murine 15-PGDH (provided in-house), and β-actin (MilliporeSigma, A5441).

### Histological and IHC analysis.

Animals were harvested via CO_2_ inhalation followed by cervical dislocation. Whole spleens or tibial BM plugs from mice and BM biopsies from human donors were fixed in 10% neutral buffered formalin. Samples were transferred to PBS and shipped to HistoWiz, where they were embedded in paraffin and sectioned at 4 μm. IHC was performed according to HistoWiz protocols (https://home.histowiz.com/faq/). HistoWiz defines their standard methods as the use of a Bond Rx autostainer (Leica Biosystems) with enzyme treatment using standard protocols, and detection via Bond Polymer Refine Detection (Leica Biosystems) according to the manufacturer’s protocol. Anti–15-PGDH staining was performed using a commercially available antibody (Abcam, EPR14332-19, catalog ab187161). Whole-slide scanning (×40) was performed on an Aperio AT2 (Leica Biosystems).

### Measurement of 15-PGDH enzymatic activity.

Splenic lysates were prepared using the Precellys 24 homogenizer, in a lysis buffer containing 50 mM Tris HCl, 0.1 mM DTT, and 0.1 mM EDTA. BM was flushed, pelleted, and lysed using the same buffer, with sonication. Enzymatic activity was measured by following the transfer of tritium from a tritiated PGE_2_ substrate to glutamate by coupling 15-PGDH to glutamate dehydrogenase (53). Activity was expressed as cpm, per mg total protein assayed.

### BMT.

Mice were exposed to 10 Gy total body irradiation from a cesium source, followed immediately by administration of PGDHi or vehicle control. Sixteen to 18 hours later, mice received 1 × 10^6^ whole BM cells by retroorbital injection, followed immediately by a second i.p. administration of PGDHi or vehicle control. Recipients continued to receive twice daily i.p. administration of PGDHi or vehicle.

### Complete blood count analysis.

Peripheral blood was collected into Microtainer EDTA tubes (Becton-Dickinson) by submandibular cheek puncture. Blood counts were analyzed using a Hemavet 950 FS hematology analyzer.

### Quantification of HSPCs and splenic cell types.

BM cells were obtained by flushing hind limb bones, and splenocytes were obtained by mincing spleens. Cellularity was measured after RBC lysis. Cells were stained with antibodies against CD45R/B220 (RA3-6B2), CD11b (M1/70), CD3ε (500A2), Ly6G and Ly6C (RB6-8C5), TER-119 (TER-119), Ly-6A/E (D7), CD117 (2B8), F4/80 (Cl:A3-1), CD61 (2C9.G2), Fcer1a (MAR-1), CD45.1 (A20), and CD45.2 (104; all obtained from BioLegend with the exception of anti-F4/80, which was obtained from Bio-Rad), and data were acquired on an LSR II flow cytometer (BD Biosciences). Analysis was performed on FlowJo software (TreeStar).

### Cell separation.

Single-cell suspensions were generated from spleen and marrows. Cells were isolated by surface marker expression using Miltenyi Biotec microbead kits and LS column separation according to the manufacturer’s instructions. 15-PGDH enzymatic activity was measured in cell fractions, or in unfractionated splenocytes or marrow cells, as described above and previously reported (14).

### RNA extraction and quantitative PCR.

CD45^+^ splenocytes or CD3ε-depleted and B220-depleted BM cells and splenocytes were isolated, as described above. Cells were lysed and RNA was extracted using the RNeasy Mini Kit (QIAGEN) with on-column DNase treatment, according to the manufacturer’s protocol. cDNA was synthesized using the PrimeScript RT Reagent Kit (Takara) following the manufacturer’s instructions. Real-time PCR (RT-PCR) measurement was performed in a 20 μL reaction containing 1 μL cDNA template and a 1:20 dilution of primer/probe with 1× Accuris Taq DNA polymerase. Samples were run on a CFX96 optical module (Bio-Rad). Thermal cycling conditions were 95°C for 3 minutes, followed by 50 cycles at 95°C for 15 seconds and 60°C for 1 minute. Murine probe/primer sets for all genes assayed were obtained from Life Technologies, Thermo Fisher Scientific, and were as follows: *B2m* Mm00437762_m1, *Ptger1* Mm00443098_g1, *Ptger2* Mm00436051_m1, *Ptger3* Mm01316856_m1, *Ptger4* Mm00436053_m1, *Actb* Mm02619580_g1, *Vcam1* Mm01320970_m1, *Crem* Mm04336053_g1, *Spp1* Mm00436767_m1, *Jag1* Mm00496902_m1, *Kitl* Mm00442972_m1, *Cxcr4* Mm01996749_s1, *Ackr1* Mm00515642_g1, *Gata1* Mm01352636_m1, *Pf4* Mm00451315_g1, *Fgf1* Mm00438906_m1, and *Cxcl12* Mm00445553_m1. For each reverse transcription reaction, Cq values were determined as the average values obtained from 3 independent RT-PCR reactions.

### Splenocyte transplantation.

Donor mice were treated for 5 days with twice daily i.p. administration of PGDHi or vehicle control. Two hours after the ninth administration, mice were sacrificed, spleens were dissected, and a single-cell suspension was generated. For noncompetitive splenocyte transplants, recipient mice were conditioned with 10 Gy irradiation 20 hours prior to the transplantation of 2 × 10^6^ splenocytes by retroorbital injection. For competitive splenocyte transplants, recipient mice were conditioned as described above and received 5 × 10^6^ splenocytes from mice treated as described above, plus 5 × 10^5^ BM cells from untreated CD45.1-expressing (B6.SJL-*Ptprc^a^ Pepc^b^*/BoyJ) mice.

### BM homing analysis.

BM was labeled with 5 μM CellTrace CFSE, and 10 × 10^6^ cells were transplanted into recipient mice that had been treated for 5 days with PGDHi or vehicle control and conditioned with 10 Gy total body irradiation 12 hours prior to transplant. Sixteen hours after transplant, mice were sacrificed, and CFSE^+^ cells were quantified in the spleen and BM flow cytometrically.

### Statistics.

All values were tabulated graphically with error bars corresponding to standard error of the means. Analysis was performed using GraphPad Prism software. Unpaired 2-tailed Student’s *t* test was used to compare groups, unless otherwise noted. For peripheral blood recovery kinetic analysis, 2-way ANOVA was used to test the effect of drug treatment. A *P* value less than 0.05 was considered statistically significant.

### Study approval.

Animals were housed in the American Association for Accreditation of Laboratory Animal Care–accredited facilities of the Case Western Reserve University (CWRU) School of Medicine. Husbandry and experimental procedures were approved by the Case Western Reserve University IACUC in accordance with approved IACUC protocols 2013-0182 and 2019-0065. Deidentified adult BM aspirates were obtained from the CWRU Hematopoietic Biorepository with permission from the IRB. Human BM aspirates were depleted of RBCs prior to cell fractionation. The human BM biopsy used for 15-PGDH IHC staining was derived from a 30-year-old female volunteer in accordance with CWRU IRB protocols.

## Author contributions

JNPS conceived and designed the study, collected and interpreted the data, and wrote the manuscript. DMD conceived and designed the study and interpreted the data. KFC and collected the data. MJC conceived and designed the study. MIA and JMR performed chemical compound purification and quality control. SLG conceived and designed the study and interpreted the data. SDM conceived and designed the study, interpreted the data, and wrote the manuscript. ABD conceived and designed the study, interpreted the data, wrote the manuscript, and provided final approval of the manuscript.

## Supplementary Material

Supplemental data

## Figures and Tables

**Table 1:**
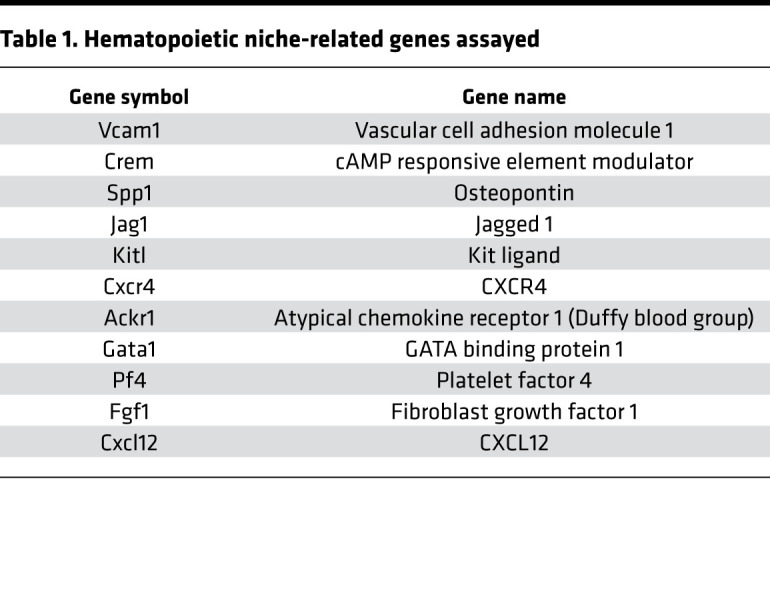
Hematopoietic niche-related genes assayed

## Version 1 02/18/2021

In-Press Preview

## Version 2 03/22/2021

Electronic publication
