# Supplementary material for: 15-PGDH inhibition activates the splenic niche to promote hematopoietic regeneration
Source: JCI Insight. 2021 Mar 22;6(6):e143658. doi: 10.1172/jci.insight.143658 (PMC8026178; doi:10.1172/jci.insight.143658)
Supplement: Supplemental data [file jciinsight-6-143658-s042.pdf]

15-PGDH Inhibition Activates the Splenic Niche to Promote Hematopoietic Regeneration

Julianne N.P. Smith[1], Dawn M. Dawson[1], Kelsey F. Christo[1], Alvin P. Jogasuria[1], Mark J. Cameron[1], Monika I. Antczak[3], Joseph M. Ready[3,4], Stanton L. Gerson[1,2], Sanford D. Markowitz[1,2], Amar B. Desai[1]

SUPPLEMENTAL MATERIAL

Supplemental Figures: 7

Supplemental Tables: 0

Supplemental References: 0

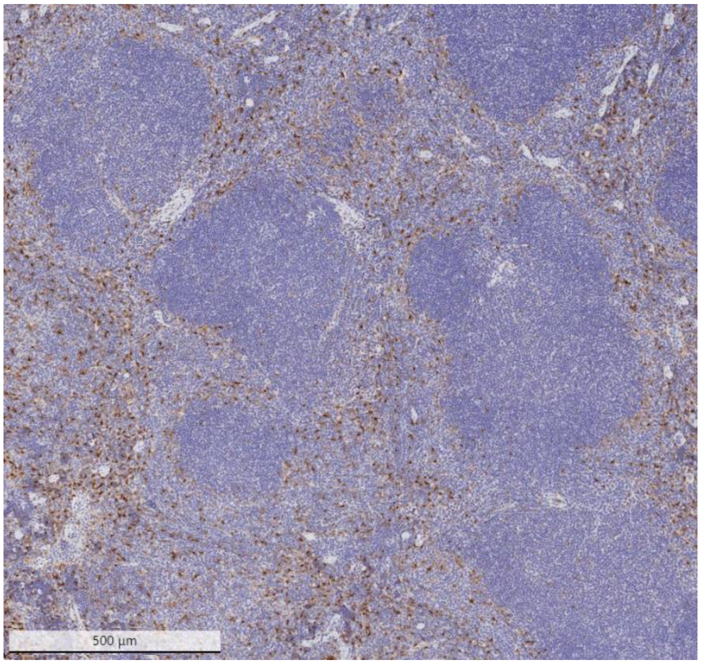

Supplementary Figure 1. 15-PGDH is expressed in the splenic red pulp. Representative image of 15-PGDH staining in the murine spleen at 5X magnification. Scale bar represents 500um. Two independent experiments of N = 2 mice per experiment.

WBCs (K cells/uL)

	D7	D12	D16	D20
Intact- Vehicle	0.453	0.947	1.765	2.460
Intact- PGDHi	0.723	1.492	2.785	3.396
Splenectomized- Vehicle	0.556	1.209	3.007	2.620
Splenectomized- PGDHi	0.548	1.212	2.805	2.855

NE (K cells/uL)

	D7	D12	D16	D20
Intact- Vehicle	0.148	0.456	0.589	0.754
Intact- PGDHi	0.260	0.935	1.129	1.170
Splenectomized- Vehicle	0.215	0.432	0.849	0.757
Splenectomized- PGDHi	0.265	0.467	0.869	0.874

PLT (K cells/uL)

	D7	D12	D16	D20
Intact- Vehicle	117	312	307	358
Intact- PGDHi	170	390	414	443
Splenectomized- Vehicle	135	312	442	439
Splenectomized- PGDHi	190	344	453	496

Supplementary Figure 2. Mean peripheral blood cell values in splenectomized versus intact Vehicle- and PGDHi-treated mice. Complete blood counts were measured at days 7, 12, 16, and 20 post-bone marrow transplant. N = 12-15 mice/group. Data represent the mean values for indicated cell types and timepoints.

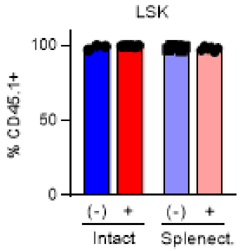

Supplementary Figure 3. LSKs in splenectomized and intact transplant-recipients are donor-derived. Quantification of percent CD45.1+ Lineage- Sca1+ c-Kit+ (LSK) cells in the marrow of intact and splenectomized, treated with Veh and PGDHi, 20 days post-transplant. N = 3-6 mice/group. Error bars represent SEM.

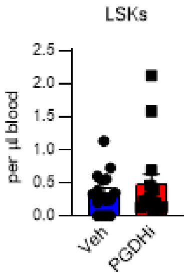

Supplementary Figure 4. PGDHi does not mobilize HSPCs. Quantification of Lineage- Sca1+ c-Kit+ cells per ul peripheral blood derived from mice treated 5 days with Veh or PGDHi. N = 15-16 mice/group. Error bars represent SEM.

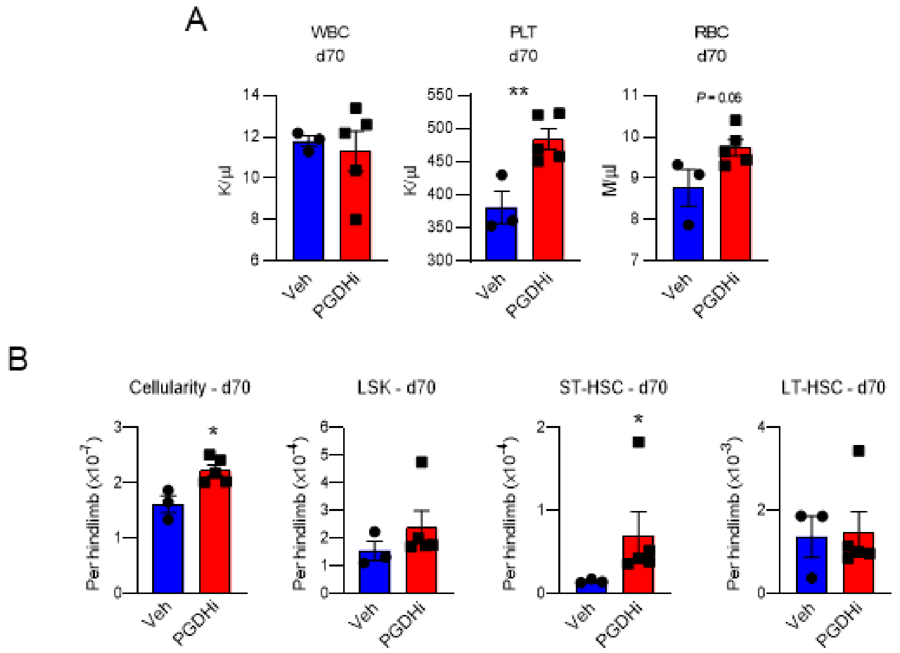

Supplementary Figure 5. PGDHi confers sustained BM engraftment capacity to transplanted splenocytes. A. Complete blood count analysis of white blood cells (WBCs), platelets (PLT), and red blood cells (RBC) in recipient mice 70 days post-transplantation of splenocytes from vehicle (Veh)- and PGDHi-treated donors. **B.** Quantification of BM cellularity, Lin- Sca1+ cKit+ (LSK), CD48- CD150- LSK (ST-HSC), and CD48- CD150+ LSK (LT-HSC) per hindlimb of recipient mice. N = 3-5 mice/group. Error bars represent SEM. Statistical analysis by Student's t-test.

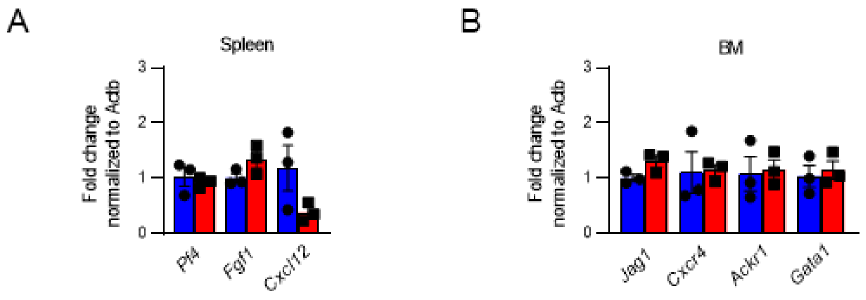

Supplementary Figure 6. Expression of additional hematopoietic niche-related genes in the BM and spleen of 5 day PGDHi-treated mice. Relative expression of the indicated genes in lymphoid-depleted cells from the spleen (**A**) and BM (**B**) of vehicle (Veh; blue) and PGDHi (red) mice. N = 3 mice/group. Error bars represent SEM.

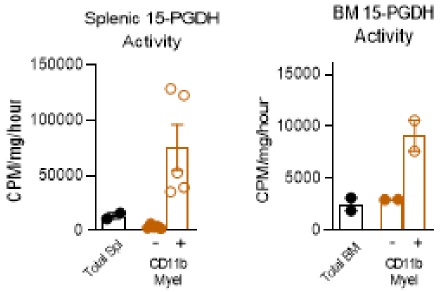

Supplementary Figure 7. Quantification of 15-PGDH enzymatic activity in splenic and bone marrow myeloid cells. Counts expressed as counts per minute (CPM) per mg total protein in total, CD11b- and CDll1b+ cells from the Spleen and BM of mice. N = 5 mice for splenic analysis and 2 mice for BM analysis. Error bars represent SEM.
